# Supplementary material for: On Associations between Fear-Induced Aggression, Bdnf Transcripts, and Serotonin Receptors in the Brains of Norway Rats: An Influence of Antiaggressive Drug TC-2153
Source: Int J Mol Sci. 2023 Jan 4;24(2):983. doi: 10.3390/ijms24020983 (PMC9867021; doi:10.3390/ijms24020983)
Supplement: Supplementary file 1 [file ijms-24-00983-s001.zip › ijms-2089554-supplementary.pdf]

Table S1. F and p values obtained in the statistical analysis (ANOVA) of acute administration of vehicle (control) or TC-2153 at the dose 10 or 20 mg/kg on mRNA expression of *Bdnf* regulatory exons I–VIII and coding (common) IX exon in the cortex of aggressive and tame rats.

Exon	Genotype effect		Drug effect		Genotype × drug interaction	
Common exon IX	$F_{1,36} = 1.52$	$p > 0.05$	$F_{2,36} < 1$		$F_{2,36} = 2.62$	$p > 0.05$
I	$F_{1,36} = 96.99$	$p < 0.001$	$F_{2,36} < 1$		$F_{2,36} < 1$	
II	$F_{1,35} = 51.35$	$p < 0.001$	$F_{2,35} = 2.22$	$p > 0.05$	$F_{2,35} = 3.16$	$p > 0.05$
III	$F_{1,35} = 11.15$	$p < 0.01$	$F_{2,35} = 1.47$	$p > 0.05$	$F_{2,35} < 1$	
IV	$F_{1,36} < 1$		$F_{2,36} = 1.5$	$p > 0.05$	$F_{2,36} = 1.09$	$p > 0.05$
V	$F_{1,35} = 19.57$	$p < 0.001$	$F_{2,35} < 1$		$F_{2,35} < 1$	
VI	$F_{1,32} < 1$		$F_{2,32} = 1.39$	$p > 0.05$	$F_{2,32} = 2.22$	$p > 0.05$
VII	$F_{1,35} = 10.77$	$p < 0.01$	$F_{2,35} = 3.02$	$p > 0.05$	$F_{2,35} < 1$	
VIII	$F_{1,35} = 9.33$	$p < 0.01$	$F_{2,35} = 1.39$	$p > 0.05$	$F_{2,35} < 1$	

Table S2. F and p values obtained in the statistical analysis (ANOVA) of acute administration of vehicle (control) or TC-2153 at 10 or 20 mg/kg on mRNA expression of *Bdnf* regulatory exons I–VIII and coding (common) IX exon in the hippocampus of aggressive and tame rats.

Exon	Genotype effect		Drug effect		Genotype × drug interaction	
Common exon IX	$F_{1,34} = 1.15$	$p > 0.05$	$F_{2,34} < 1$		$F_{2,34} < 1$	
I	$F_{1,30} = 8.15$	$p < 0.01$	$F_{2,30} < 1$		$F_{2,30} = 3.09$	$p > 0.05$
II	$F_{1,33} = 19.01$	$p < 0.001$	$F_{2,33} < 1$		$F_{2,33} < 1$	
III	$F_{1,36} = 27.88$	$p < 0.001$	$F_{2,36} = 1.92$	$p > 0.05$	$F_{2,36} < 1$	
IV	$F_{1,33} = 7.18$	$p < 0.05$	$F_{2,33} = 2.65$	$p > 0.05$	$F_{2,33} < 1$	
V	$F_{1,33} = 4.29$	$p < 0.05$	$F_{2,33} = 3.54$	$p < 0.05$	$F_{2,33} < 1$	
VI	$F_{1,33} = 19.62$	$p < 0.001$	$F_{2,33} = 6.21$	$p < 0.01$	$F_{2,33} < 1$	
VII	$F_{1,34} = 18.18$	$p < 0.001$	$F_{2,34} < 1$		$F_{2,34} < 1$	
VIII	$F_{1,34} = 14.56$	$p < 0.001$	$F_{2,34} = 2.50$	$p > 0.05$	$F_{2,34} < 1$	

Table S3. F and p values obtained in the statistical analysis (ANOVA) of acute administration of vehicle (control) or TC-2153 at 10 or 20 mg/kg on mRNA expression of *Bdnf* regulatory exons I–VIII and coding (common) exon IX in the hypothalamus of aggressive and tame rats.

Exon	Genotype effect		Drug effect		Genotype × drug interaction	
Common exon IX	$F_{1,35} = 11.17$	$p < 0.01$	$F_{2,35} < 1$		$F_{2,35} < 1$	
I	$F_{1,33} = 54.65$	$p < 0.001$	$F_{2,33} = 7.66$	$p < 0.01$	$F_{2,33} < 1$	
II	$F_{1,35} < 1$		$F_{2,35} = 2.20$	$p > 0.05$	$F_{2,35} = 1.94$	$p > 0.05$
III	$F_{1,35} < 1$		$F_{2,35} = 3.14$	$p > 0.05$	$F_{2,35} < 1$	
IV	$F_{1,33} < 1$		$F_{2,33} < 1$		$F_{2,33} < 1$	
V	$F_{1,35} < 1$		$F_{2,35} = 3.19$	$p = 0.053$	$F_{2,35} < 1$	
VI	$F_{1,32} < 1$		$F_{2,32} < 1$		$F_{2,32} < 1$	
VII	$F_{1,36} = 2.48$	$p > 0.05$	$F_{2,36} = 6.54$	$p < 0.05$	$F_{2,36} < 1$	
VIII	$F_{1,30} = 1.18$	$p > 0.05$	$F_{2,30} = 1.32$	$p > 0.05$	$F_{2,30} < 1$	

Table S4. F and p values obtained in the statistical analysis (ANOVA) of acute administration of vehicle (control) or TC-2153 at the dose 10 or 20 mg/kg on mRNA expression of *Bdnf* regulatory exons I–VIII and coding (common) exon IX in the midbrain of aggressive and tame rats.

Exon	Genotype effect		Drug effect		Genotype × drug interaction	
Common exon IX	$F_{1,36} = 1.99$	$p > 0.05$	$F_{2,36} = 3.18$	$p = 0.053$	$F_{2,36} < 1$	
I	$F_{1,36} = 52.76$	$p < 0.001$	$F_{2,36} = 2.00$	$p > 0.05$	$F_{1,36} < 1$	
II	$F_{1,35} = 15.95$	$p < 0.001$	$F_{2,35} = 5.36$	$p < 0.01$	$F_{2,35} < 1$	
III	$F_{1,34} = 6.36$	$p < 0.05$	$F_{2,34} < 1$		$F_{2,34} < 1$	
IV	$F_{1,35} = 4.59$	$p < 0.05$	$F_{2,35} = 7.98$	$p < 0.01$	$F_{2,35} < 1$	
V	$F_{1,35} = 7.55$	$p < 0.01$	$F_{2,35} = 4.74$	$p < 0.05$	$F_{2,35} < 1$	
VI	$F_{1,34} < 1$		$F_{2,34} < 1$		$F_{2,34} = 1.17$	$p > 0.05$
VII	$F_{1,33} < 1$		$F_{2,33} = 9.53$	$p < 0.001$	$F_{2,33} < 1$	
VIII	$F_{1,36} < 1$		$F_{2,34} = 3.82$	$p < 0.05$	$F_{2,36} < 1$	
